# Advances in nanomaterials for enhancing cGAS-STING pathway mediated anti-tumor treatment

**DOI:** 10.1016/j.mtbio.2025.102190

**Published:** 2025-08-11

**Authors:** Ruicheng Wu, Jie Wang, Dengxiong Li, Ao Li, Koo Han Yoo, Zhihong Liu, Wuran Wei, Zhipeng Wang, Dechao Feng

**Affiliations:** a Department of Urology, The First Affiliated Hospital of Zhejiang Chinese Medical University (Zhejiang Provincial Hospital of Chinese Medicine), Hangzhou, Zhejiang Province, China; b Department of Urology, Institute of Urology, West China Hospital, Sichuan University, Chengdu, 610041, China; c Division of Surgery & Interventional Science, University College London, London, W1W 7TS, UK; d Department of Pediatric Urology, Sichuan University West China Second University Hospital, Chengdu, Sichuan, China; e Department of Urology, Kyung Hee University, South Korea; f Department of Urology, Sichuan Provincial People's Hospital, University of Electronic Science and Technology of China, Chengdu, 610072, China

**Keywords:** cGAS-STING pathway, Nanomaterials, Cancer immunotherapy, Targeted delivery

## Abstract

The cGAS-STING pathway is a crucial component of innate immunity, playing a dual role in tumor biology by promoting pro-inflammatory responses and anti-tumor immunity. Nanomaterials have emerged as transformative tools to enhance the activation and therapeutic efficacy of this pathway in cancer immunotherapy. This review targets recent advancements in nanomaterials of enhancing cGAS-STING-mediated anti-tumor immune responses. These nanomaterials leverage their unique physical and chemical properties for targeted delivery, controlled release, and biodegradability, significantly improving the anti-tumor effects through synergistic activation of the cGAS-STING pathway. This review underscores the potential of nanomaterial-based cGAS-STING therapeutics for cancer treatment and identifies key research priorities to address existing limitations and propel the field forward.

## Graphical abstract

**Figure:**
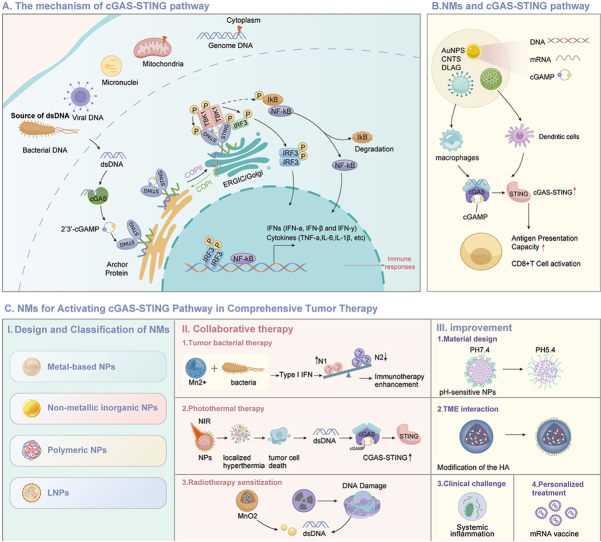

## 1 Introduction

Innate immunity serves as the body's primary defense against pathogen infections and cellular stress. It is capable of sensing both endogenous and exogenous pathogen-associated molecular patterns as well as damage-associated molecular patterns through pattern recognition receptors. This system rapidly identifies and eliminates abnormal cells, thereby inhibiting the onset of disease [1]. In tumor cells, dysfunctions in the DNA damage response (DDR), mitosis, or telomere maintenance result in the accumulation of cytoplasmic DNA. Defects in DDR primarily lead to increased mutagenesis, replication stress, and the accumulation of various forms of nucleic acid fragments that are ultimately released into the cytoplasm [2,3]. Additionally, other types of abnormal DNA, including extrachromosomal DNA, extrachromosomal telomeric repeat DNA, and mitochondrial DNA, also accumulate in tumor cells [4]. As a crucial component of innate immunity, cyclic GMP–AMP (cGAMP) synthase (cGAS) recognizes and binds to these abnormal double-stranded DNA (dsDNA) in a non-specific manner, catalyzing the production of cGAMP, which acts as a second messenger to bind and activate stimulator of interferon genes (STING) [5]. STING subsequently initiates downstream transcription programs, inducing the expression of type I interferons and pro-inflammatory cytokines through the phosphorylation of interferon regulatory factor 3 (IRF3) and TANK-binding protein kinase 1 (TBK1) [6,7]. This process promotes the expression of antigen-presenting cells (APCs), while the activation and expansion of effector T cells further assist the immune system in eliminating tumor cells. STING activation involves the activation of the Nuclear Factor kappa-light-chain-enhancer of activated B cells (NF-κB) pathway, the transcription of genes encoding pro-inflammatory cytokines in the nucleus, and under certain conditions, STING activation has been reported to induce autophagy, necroptosis, and lysosome-mediated cell death [[8], [9], [10]]. In addition to the classical cCGA-STING-TBK1/IRF3 axis, the cGAS-STING pathway also participates in the maintenance of cell homeostasis through a variety of non-canonical mechanisms. T cells can induce the accumulation of reactive oxygen species (ROS), leading to DNA fragmentation in interacting dendritic cells (DCs), and STING activates NF-κB via TRAF6 [11]. STING can directly interact with PERK kinase on endoplasmic reticulum membrane after binding to cGAMP and induce its activation, thereby mediating eIF2αS51 phosphorylation. The activation of this pathway has been confirmed to be closely related to cell senescence [12]. Recent studies have also shown that the ORF2 protein of the LINE-1 retrotransposition element has a significant reverse transcriptase activity in the cytoplasm, which can directly synthesize RNA:DNA hybrids and activate the cGAS-STING pathway, representing a non-classical innate immune activation triggered by endogenous nucleic acid replication [13]. However, tumors can achieve immune escape by inhibiting or interfering with the cGAS pathway. Overexpression of TREX1 in tumor cells may avoid the engagement of cGAS pathway by degrading cytoplasmic DNA [14]. In hypoxic circumstances, tumor cells suppress cGAS pathway activity due to JNK1/2-mediated phospho-PCK1 which interacts with cGAS and then competes for GTP binding [15]. ADSL enzyme inactivates STING through phosphorylation process that occurs under hypoxic conditions [16]. Thus, it has been proposed that the restoration or enhancement of cGAS-STING signaling could be a critical approach to overcoming the immune tolerance state.

Due to the antitumor effects elicited by the activation of the cGAS–STING pathway, numerous STING agonists, such as MIW815, have entered clinical trials in recent years to evaluate their safety and efficacy in treating solid tumors [17]. However, the clinical efficacy of STING is still limited by challenges such as poor cellular uptake, short half-life period and insufficient targeting. For example, ADU-S100 require direct intratumoral injection to achieve local immune activation due to the limited efficacy of systemic administration [18]. Multiple alternative strategies are currently available, including the use of lipid-soluble derivatives and non-CDN agonist. Loading STING agonists with positively charged liposomes can enhance targeting, but long-term use of liposomes may cause systemic toxicity, and their stability may be affected by enzymatic action, leading to premature drug release [19]. Although several non-CDN agonist have also shown promising clinical activity, including M335 and E7766, they still face the problems of limited solubility and lack of delivery specificity [20,21]. Nanomaterials (NMs) provide a modular, tunable delivery platform. Advantages of using NMs include precise control of particle size and surface charge to protect STING agonists from enzymatic degradation [22,23]. Co-delivery of adjuvants and chemotherapeutic agents can also be achieved [24]. In addition, certain nanocellulars, such as metal ionophores, have intrinsic immunostimulatory properties that further amplify antigen presentation and T-cell priming [25]. Collectively, these features make NMs well suited to fulfill the therapeutic potential of STING activation. Furthermore, NMs can integrate various therapeutic modalities, including chemotherapy, radiotherapy, and photothermal therapy (PTT), to synergistically amplify antitumor immune responses through the cGAS-STING pathway. In addition, the use of bacteria to modulate the tumor microenvironment (TME) provides a new strategy for enhancing STING-mediated antitumor therapy.

This review focuses on the latest advancements in NMs designed to enhance the antitumor effects mediated by the cGAS-STING pathway. It highlights the properties of various types of NMs and their roles in antitumor therapy, aiming to provide an overview of the current applications and future outlook of cGAS-STING-targeted nanotherapeutics.

## 2 Design and classification of nanomaterials

**Fig. 1:**
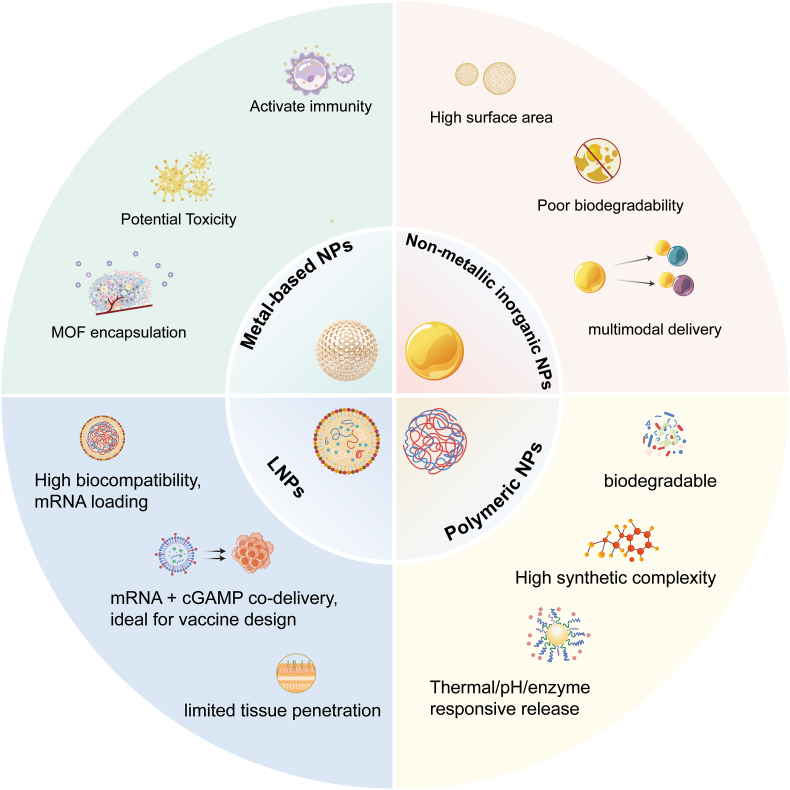
**Overview of different types of nanomaterials and their characteristics;** MOF: Metal–Organic framework; LNPs: Lipid nanoparticles; NPs: nanoparticles; cGAMP: cyclic GMP–AMP.

The key of treatment strategies around the cGAS pathway is to promote the activation of the pathway. This not only requires efficient STING agonists, but also depends on the design of delivery systems. NMs with different properties can improve the targeting of STING agonists, and even the NMs themselves can stimulate STING-related signaling. This section will illustrate how different classes of NMs support STING activation. Fig. 1 summarizes the characteristics of various materials.

### 2.1 Metal nanoparticles

Metal nanoparticles (MNPs) exhibit excellent stability and surface functionalization capabilities [26]. The distinctive surface plasmon resonance properties of these particles allow interaction with specific wavelengths of light, enabling applications in bioimaging and PTT [27,28]. MNPs also demonstrate low toxicity and good biocompatibility in both in vivo and in vitro, making them suitable for a wide range of biomedical applications [29]. Cells inherently contain various metal ions, such as those associated with the Na^+^/K^+^ pump on the cell membrane [30]. A variety of metal ions are closely related to STING pathway activation. Studies have found that the up-regulation of ferritin expression can lead to an increase in the level of Fe^2+^ in the liver, which in turn activates the STING pathway [31]. The accumulation of Fe^2+^ can lead to oxidative stress and generation of excess ROS, resulting in mitochondrial dysfunction, and mitochondrial DNA (mtDNA) released to the cytoplasm triggers the cGAS pathway [32,33]. Ag^+^ are able to bind to sulfhydryl groups in proteins and induce DNA damage [34]. In addition, Ag^+^ can induce pyroptosis and release pro-inflammatory factors, which further activate the STING pathway [35]. Ca^2+^can also affect the activation of STING. STIM1 indirectly controls the localization and activity of STING by sensing the change of endoplasmic reticulum Ca^2+^concentration [36]. Mechanical stress also leads to the release of mtDNA after mitochondrial calcium overload, triggering the activation of the STING pathway [37].

Zn^2+^ and Mn^2+^ ions are the most commonly used metal-organic framework (MOF) to activate STING pathway. Zn^2+^ activated the STING pathway by enhancing the binding ability of cGAS to dsDNA. In vitro, Zn^2+^ significantly lowered the threshold of cGAS activation, and cGAMP formation could be catalyzed by cGAS as low as 2.5 nM [38,39]. In addition, similar to other metal ions, Zn^2+^ can also promote mtDNA release and ROS production [40]. Similar to zinc ions, Mn^2+^ can bind to cGAS protein, changing its conformation and enhancing its affinity for dsDNA [41]. On the other hand, Mn^2+^ also promoted DNA release from the cells by inducing oxidative stress [32]. Structurally, Mn^2+^ can be stably encapsulated in MnO_2_ or MOF-like NMs and rapidly released in the acidic tumor microenvironment, thereby achieving targeted cGAS-STING activation [42]. Mn^2+^ also amplifies immune responses by activating TLR4-mediated signaling cascades [43,44]. Less reported ion types, such as La^3+^ and MoO_4_^2−^, have also been shown to regulate the cGAS-STING pathway in recent studies [45,46].

### 2.2 Non-metallic inorganic nanomaterials

In addition to metal-based nanocrystals, nonmetallic inorganic nanomaterials can also serve as multifunctional platforms for the activation of the STING pathway. Mesoporous silica NMs (MSNs) have a controllable mesoporous structure with a high specific surface area and pore volume, which can effectively load a variety of drugs and types of functional molecules to achieve multimodal combined delivery. MSNs not only efficiently release cGAMP in the cytoplasm to activate STING, but also induce mitochondrial damage to promote mtDNA leakage, thereby enhancing cGAS recognition and downstream signal amplification. The good surface modifiability of carbon-based materials can also be used to load STING agonists.

### 2.3 Polymeric nanomaterials

Polymeric nanomaterials provide a tunable platform for STING agonist delivery. Polymeric micelles (PMs) are typically small size (10–100 nm), which allows their increased prisence in tumor tissues through enhanced permeability and retention effects [47]. The hydrogen-bonded micellar CUH-cGAMP developed by Park et al. releases cGAMP within cells in a controlled manner, effectively maintaining STING activation and inducing anti-tumor immune responses [48]. The PMs can also be used as a multi-component delivery system, and its temperature responsiveness can cause the structure to collapse through thermal effects, so as to accurately release the internal drug [49]. Polymersomes are vesicles like organelles that have a membrane structure of bilayer. It also has excellent stability and longer cycle time. The modification of polyethylene glycol can increase the circulation time and stability of NMs, preventing their rapid clearance by the immune system in the bloodstream, thus improving drug bioavailability [50]. Along with that, the biodegradable and biocompatible polymers have good safety. For instance, Polylactic-glycolic acid (PLGA) NMs exhibit excellent biodegradability, decomposing into lactic acid and glycolic acid within the body without causing toxic reactions [51]. Target delivery or controlled release can be achieved through the design of the system on the basis of stimuli such as pH, temperature and enzyme [52]. Polymersomes not only serve as delivery platforms to enhance STING agonist activity, but also can be exploited as novel STING agonists themselves [53,54]. In addition, polymer nanogels—nanoscale hydrogel particles formed by cross-linked polymer networks—offer high drug-loading capacity, excellent colloidal stability, and stimuli-responsive release behaviors, making them ideal candidates for intracellular delivery of STING agonists [55].

### 2.4 Lipid Nanoparticles

Lipid Nanoparticles (LNPs) are nanoparticle structures formed by self-assembly of lipid molecules. The lipid composition makes LNPs have excellent biocompatibility and efficient hydrophilic drug loading ability, which is suitable for encapsulating hydrophilic molecules such as mRNA and cGAMP [56]. It is particularly suitable for the delivery of mRNA that is unstable, degradable, and needs to be expressed in the cytoplasm to induce immune responses against tumor-associated antigens [22]. One of the core designs of LNPs is the use of ionized lipids to trigger membrane structure rupture in the acidic endosomal environment and improve the endosomal escape efficiency [57]. In addition, the flexible structure of LNPs gives them the ability of rapid response and release, which avoids the problem of delayed drug effect due to high stability of the drug loading system [58].

## 3 Nanomaterials for activating cGAS-STING pathway in comprehensive tumor therapy

Advancements of nanocarrier design have significantly improved the efficacy of cGAS-STING pathway activation. Being a carrier of STING agonists, some nanomaterials themselves, especially nanoparticles containing bioactive metal ions, can also be involved in pathway activation directly. Moreover, combining nano-platforms with techniques such as photothermal therapy, radiotherapy or gas therapy can create a synergistic therapeutic system that can not only induce innate immunity but also modulate the tumor microenvironment and has a broader application for immunotherapy.

In tumor immunotherapy, the delivery carrier of STING agonists plays a crucial role in activating the cGAS-STING pathway. Researchers have developed various NMs carriers to enhance the stability, targeting, and delivery efficiency of STING agonists, thereby more effectively stimulating anti-tumor immune responses. Dane et al. [59] designed a PEGylated nanoparticle that avoids premature clearance by the immune system through PEG modification, thus prolonging its circulation time in the body. This approach not only increases the stability of STING agonists but also facilitates their effective accumulation at the tumor site through enhanced tumor penetration, significantly improving local immune activation. Zheng et al. [60] utilized polymersomes as delivery vehicles for the STING agonist cGAMP. The double-layer structure of these vesicles effectively protects cGAMP from enzymatic degradation in the body and enables its slow release in the cytoplasm, thereby improving the activation intensity and duration of the STING pathway. There are also explorations into utilizing the microenvironment to enhance drug delivery. Xiao et al. [61] developed a pH- and enzyme-responsive nanoparticle carrier that leverages the acidic properties of the lymph node microenvironment for precise drug release. Under physiological conditions in normal tissues, STING agonists are encapsulated within nanoparticles; however, they are released in the acidic environment of the lymph nodes, where they directly activate dendritic cell and T cells, while also associating with activated T cells via PD-1 binding. At the cellular level, the tumor-homing capability of PD-1+ T cells facilitates effective drug delivery to tumor tissues. STING is localized exclusively to the cytoplasm. The researchers employed biodegradable poly (beta-amino ester) nanoparticles to deliver CND directly into the cytoplasm. This approach ensures that when the extracytoplasmic CND concentration is low, cGAS-STING remains activated and demonstrates a robust response to melanoma tumors in vivo [62]. Metal ions can directly bind to cGAS, altering its conformation and increasing its affinity for cytosolic DNA. This interaction facilitates the recognition of free DNA by cGAS and significantly enhances the efficiency of cGAMP synthesis. Additionally, some studies have indicated that hyaluronic acid modification can enhance the biocompatibility of nanoparticles and facilitate tumor-specific targeting by binding to the CD44 receptor on tumor cells [63]. When utilized as a delivery system, peptide nanotubes exhibit high drug encapsulation efficiency and stability under physiological conditions. This characteristic enables the gradual release of STING agonists within the tumor microenvironment, thereby facilitating effective immune activation [64]. Hydrogels serve as excellent carriers for STING agonists. Cheng et al. [65] designed a silk fibroin hydrogel for use as an in situ vaccine. The incorporation of cGAMP nanoparticles with a core-shell structure optimized the delivery efficacy and utilized immunogenic cell death (ICD) inducers to enhance and prolong STING activation, thereby inhibiting tumor progression across various immune tumor models.

NMs assembled with manganese ions as the core are delivered to the tumor microenvironment for degradation and the subsequent release of Mn^2+^, which not only activates the STING pathway but also serves as an immune-stimulating factor to further amplify the anti-tumor immune response [[66], [67], [68]]. For instance, He et al. [69] designed a manganese-phenol nanoparticle decorated with lipopolysaccharide. The natural polyphenol coordinates with Mn^2+^ and immediately adheres to the surface of individual cancer cells, resulting in a nano-free cloak that encapsulates tumor antigens. Subsequent modification with lipopolysaccharide induces dendritic cell internalization, during which Mn^2+^ ions are released into the cytosol, further promoting the activation of the STING pathway. Additionally, some studies have found that iron ions can generate reactive oxygen species in tumor cells, leading to the leakage of DNA into the cytoplasm and stimulating cGAS. Zinc ions can also enhance cGAS enzyme activity by promoting cGAS-DNA phase separation [49,70]. In addition to these common metal ions, mixed nanoformulations containing lanthanum ions have also been shown to significantly enhance DC maturation and facilitate the infiltration of CD8^+^ T cells into tumors, thereby inhibiting tumor growth [45]. In the TME, NMs can induce ferroptosis, leading to the release of oxidative damage molecules and cytosolic DNA, which serve as immune signals to activate the cGAS-STING pathway, thereby amplifying the anti-tumor immune response. The nanomolecules designed by Wang et al. [71] initiate Fenton and Fenton-like reactions in the tumor microenvironment by releasing Mn^2+^ and Fe^3+^, resulting in the generation of a substantial amount of reactive oxygen species. This process triggers lipid peroxidation and glutathione depletion, which in turn inhibits GPX4 expression and induces ferroptosis. Ferroptosis-induced immunogenic cell death (ICD) is also facilitated by the release of HMGB1 and surface-exposed calreticulin, further activating anti-tumor immunity. This process enhances immune-driven ferroptosis through the cGAS-STING pathway, creating a positive feedback loop that reinforces anti-tumor immunity [72]. Various metal ions are integrated into NMs to activate the STING pathway while inducing ferroptosis. Mixed NMs containing calcium and manganese can lead to calcium overload in mitochondria, thereby exacerbating oxidative stress [73]. Molybdenum ions, functioning as anions, can also serve as cGAS-STING agonists. High-valent molybdenum and manganese in molybdenum-manganese nanoparticles induce ferroptosis and elicit tumor-specific immune responses while simultaneously activating cGAS-STING to further enhance this immune response [46]. Consequently, double metal oxide nanostructures demonstrate promising potential in tumor immunotherapy. Shi et al. [74] developed magnetic nanoparticles based on manganese ferrite. The application of a magnetic field facilitates a specific targeting effect by enabling the binding of RGD, which selectively attaches to tumor cells expressing the αvβ_3_ integrin. The surface modification of these NMs endows them with exceptional tumor targeting capabilities. Manganese carbonyl, modified with PEG and RGD, can effectively decompose in the presence of H_2_O_2_ within the tumor microenvironment, leading to the release of Mn^2+^ and carbon monoxide and achieving pyrolysis and dual activation of the cGAS-STING pathway [75].

Collaborative therapy integrates radiotherapy, immunotherapy, PTT and other modalities, utilizing NMs to precisely deliver and activate the cGAS-STING pathway. Biomineralized MnO_2_ nanoparticles address the prevalent issue of hypoxia in radiotherapy by converting H_2_O_2_ within the tumor microenvironment into oxygen, thereby improving the production of radiation-induced dsDNA breaks and enhancing radiosensitivity [76,77]. It also releases Mn^2+^ to participate in the downstream activation of cGAS, and induces ICD to promote antigen release and synergistically amplify the immune effect [78]. Yi et al. [79] developed a hybrid nanoadjuvant system (Tpp-Met@MnO_2_@Alb) that combines a metformin derivative (Tpp-Met), MnO_2_, and albumin as a biocompatible carrier. Tpp-Met can inhibit oxidative phosphorylation to reduce tumor hypoxia and improve radiosensitivity, and down-regulate the expression of PD-L1 and TGF-β1 in the membrane and cytoplasm. MnO_2_ releases Mn^2+^ in the acidic tumor environment to activate the STING pathway. Tumor bacterial therapy is a strategy that employs bacteria or their products to selectively target tumor tissues for therapeutic purposes [80]. When colonizing the tumor, Salmonella polarizes neutrophils to the N2 phenotype. In contrast, Mn^2+^ promotes the secretion of type I interferon, which induces the transformation of neutrophils from the pro-tumor N2 phenotype to the anti-tumor N1 phenotype, thus constructing a combined immune activation pathway of "bacteria + STING" [81]. Additionally, extracellular DNA secreted by bacteria can activate the cGAS-STING pathway, contributing to synergistic tumor immunotherapy [82]. PTT mainly relies on photosensitive materials to absorb near-infrared light and convert it into local heat to destroy tumor tissues. The oxidation-responsive metal-organic framework nanoparticles developed by Zhou et al. [83] generate singlet oxygen to induce tumor cell death while releasing the cGAS-STING agonist SR-717 to enhance both local and systemic immune responses. Chen et al. [84] constructed a chitosan hydrogel nanosystem that integrates the photothermal agent indocyanine green with the STING agonist dimethylxanthenone-4-acetic acid to destroy tumor tissue through localized heating under light exposure and to release cytoplasmic DNA for cGAS activation. Xia et al. [85] proposed a manganese (III)-doped photothermal nanosystem that employs the photothermal effect to induce tumor cells to release substantial amounts of cytosolic DNA. Concurrently, this system enhances the binding efficiency of cGAS and DNA by releasing manganese ions, significantly improving the activation of the STING pathway. Zhao et al. [86] constructed an integrated nanoplatform (AIEgen@BNN6) combining an aggregation-induced luminescence molecule (AIEgen) with gas therapy, which chemically bonded the photosensitizer TPA-BDTO-NH_2_ to the NO donor molecule BNN6 and encapsulated in nanoparticles. The AIEgen fraction was subjected to 660 nm laser irradiation to generate ROS, which induced ICD and cytosolic dsDNA release. BNN6 releases nitric oxide under heat/light stimulation, which further aggravates DNA damage and promotes cGAS expression and STING pathway activation.

**Fig. 2:**
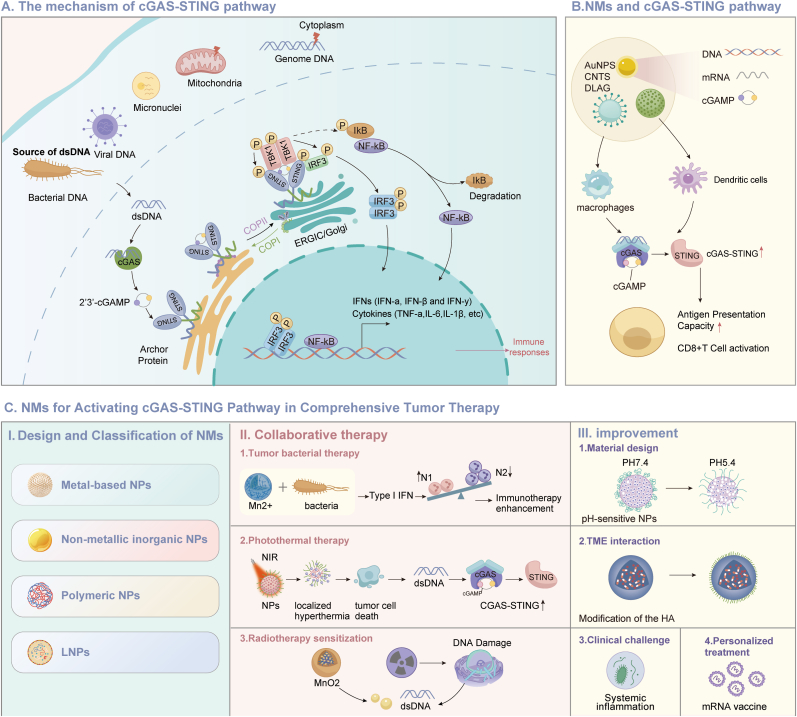
**Nanomaterial-based strategies to activate the cGAS–STING pathway for tumor immunotherapy. (A)** The basic mechanism of the cGAS-STING pathway; **(B)** The principle of NMs in activating the cGAS-STING pathway; **(C)** Specific therapeutic strategies leveraging the cGAS-STING pathway, showcasing diverse approaches for cancer immunotherapy enabled by NMs. cGAS: Cyclic GMP–AMP synthase; dsDNA: Double-stranded DNA; STING: Stimulator of interferon genes; TBK1: TANK-binding protein kinase 1; IRF3: Interferon regulatory factor 3; NF-κB= Kappa-light-chain-enhancer of activated B cells; NMs: Nanomaterials; MNPs: Metal nanoparticles; CNTs: Carbon nanotubes; PNPs: Polymeric nanoparticles.

NMs offer a variety of strategies to activate the cGAS-STING pathway, leveraging their physicochemical properties and design optimizations to exert anti-tumor effects. The integration of NMs with multimodal treatments, such as PTT and radiotherapy, enhances anti-tumor immune responses by remodeling the TME, thereby facilitating the transformation of 'cold' tumors into 'hot' tumors [49,87]. Future developments should prioritize the combination of the high efficiency of NMs with clinical practicality, further enhancing the overall efficacy of tumor immunotherapy through the integration of intelligent delivery systems, microenvironment regulation, and collaborative multimodal treatment. Table 1 highlights the key references underpinning this review, while Fig. 2 visually summarizes its core content.

**Table 1:** Summary of core literature discussed in this review.

PMID	Elements	Cancer type	Cell lines	Animal Models	Results
38229577	Triphenylphosphine and MnO_2_	Bladder cancer and breast cancer	MB49, 4T1 and 5637	BALB/c mice	Inhibition of PD-L1 and TGF-β1 and activation of cGAS-STING pathway
38414097	*Escherichia coli* and Mn^2+^	Melanoma and liver cancer	DC2.4, B16F10 and VX2	Male mice and rabbits	Mn^2+^ enhances the sensitivity of cGAS to extracellular DNA secreted by bacteria and activates the CGAS-STING pathway
38639726	diABZI-C_2_-NH_2_ and GSK-3484862	Breast cancer	T41 and CTLL-2	BALB/c mice.	The nanomedicine releases STING agonists in the acidic environment of the lymph nodes to activate DC and T cells
38663684	MnFe_2_O_4_@NaGdF4	Cervical cancer	Hela cells	Balb/c mice	Overcome the immunoheterogeneous microenvironment to increase the efficacy of iron death
38225710	Zinc oxide and doxorubicin	Breast cancer	/	BALB/c mice.	Activation of STING pathway leads to dendritic cell maturation and promotes immune response
38660408	MnFe_2_O_4_	Melanoma, colon and breast cancer	/	Male mice	Promote tumor targeted imaging and activate STING pathway
38734282	MnO_2_ and oxaliplatin	Colon cancer	L929 and CT26	Male mice	The combined action of oxaliplatin and Mn^2+^ activated the cGAS-STING pathway

39068474	MnO_2_	Breast cancer	/	BALB/c mice	MnO_2_ nanoparticles were used in combination with Salmonella to enhance the antitumor effect
38251849	Oligodeoxynucleotides and Mn^2+^	Breast cancer	NIH 3T3, RAW 264.7, and 4T1	4T1 tumor-bearing mice	The nanosheets activated toll-like receptor 9 and STING pathways in acidic TME and endosomal compartments
37601278	Mn^2+^ and MoO_4_^2^^−^	Melanoma, colon and breast cancer	93-Dual™ mSTING, HUVESs, DC2.4, CT26, 4T1 and B16F10	CT26, 4T1 and B16F10 tumor-bearing mice	MoO_4_^2-^ and Mn^2+^ induce iron death and activate the cGAS-STING pathway
38302821	Polyphenols and Mn2+	Melanoma	B16F10	C57BL/6 mice	Mn^2+^ adhered to tumor cells and released and activated STING pathways in the cytoplasm
36848115	MnO_2_	Colon cancer	HUVEC, DC2.4 and CT26	BALB/c mice	Activation of STING pathway simultaneously promotes intratumoral invasion of CTL and initiates systemic anti-tumor response
38264682	Carbon monoxide and MnO2	Breast cancer	HEK293 and 4T1	BALB/c mice	Following the degradation of the nanosystem in the TME, MnO_2_ activated the STING pathway, while the controlled release of CO further stimulated tumor immunity
35386324	CDNs	Melanoma	B16F10	B16F10 tumor-bearing mice	Tumor retention and cytoplasmic delivery of CDN were enhanced to enhance STING pathway activation
36144927	MnO_2_	Lung cancer	A549, PC9, H520 and LLC	C57BL/6 mice	Alleviating tumor hypoxia and activating cGAS-STING pathway synergistic immune response
35981098	Manganese zinc sulfide nanoparticles	Melanoma	B16F10	C57BL/6 mice	Mn^2+^-mediated CDT promotes ROS production through photothermal triggering, maximizes tumor ICD, and activates STING pathway
35606429	CDNs	Colon and breast cancer	MC38, TC-1, 4T1-Luc	C57BL/6 mice, BALB/c mice and STING-deficient Goldenticket mice	Demonstrated more effective tumor permeability by exposing cells to STING agonists
33448035	Chitosan	Melanoma	B16F10	Mice with Luc-B16F10 lung metastasis	The nanodelivery was assembled with aPD-L1 to synergistically activate STING pathway and ICB therapy
32484320	Mn_3_O_4_ and doxorubicin	Melanoma and breast cancer	B16F10, 4T1 and RAW 264.7	C57BL/6 mice	STING-mediated immunotherapy combined with chemotherapy inhibits tumor growth
29127039	CDNs and poly (beta-amino ester)	Melanoma	THP1, B16-F1	C57BL/6 mice	Delivery of the CDNs into the cytoplasm activates the immune response

## 4 Perspective

The integration of NMs with the cGAS-STING pathway presents transformative possibilities for tumor immunotherapy. Despite notable advancements, substantial challenges persist in areas such as material design, biocompatibility and safety, scalability, and the interaction of therapeutic modalities. Addressing these challenges necessitates focused efforts to facilitate the practical and effective application of these advanced therapies.

The physical and chemical properties of NMs directly influence their in vivo behavior, tissue distribution, and toxicity. Particle size is a critical factor affecting the distribution and cellular uptake of NMs. Smaller NMs (50–200 nm) typically enter cells via endocytosis, making them suitable for targeting specific receptors [88]. In contrast, larger particles (above 200 nm) primarily rely on macropinocytosis and tend to be taken up non-specifically [89]. There exists a complex relationship between particle size and the rate of cellular uptake. Notably, the intracellular accumulation of smaller gold NMs is significantly higher than that of larger one [90]. However, research has shown that the cellular uptake efficiency of certain polymer nanoparticles can increase with particle size, with 900 nm particles exhibiting the highest phagocytosis efficiency in macrophages [91]. In addition, softer nanoparticles are more likely to enter cells by caveolin-mediated endocytosis than particles with higher stiffness [92]. In addition, the particle size also affects the tissue distribution of nanomaterials. Xia et al. [93] found that with increasing particle size (5–50 nm), 50 nm AuNPs had the longest blood circulation time and the highest distribution in the liver and spleen. The structural shell structure can not only increase the stability of NMs, but also increase the targeting ability [94]. For example, the core-shell NMs modified by T7 peptide can efficiently deliver siRNA to the tumor site through receptor-mediated endocytosis [95]. The degradability of nanomaterials is closely linked to their safety profiles. Inorganic NMs typically exhibit low acute toxicity due to their high chemical stability. However, prolonged exposure may result in the accumulation of metal ions in organs, potentially leading to chronic inflammation or organ dysfunction [96]. Degradation products of organic NMs may also induce local tissue damage, yet they often enhance biocompatibility by introducing functional groups on the material's surface [97]. The immune recognition and response induced by NMs represent a risk factor in the assessment of biological safety. While biomimetic strategies, such as PEG modification and cell membrane camouflage, can extend in vivo circulation times and reduce the immunogenicity of NMs, there remains a risk of activating the STING pathway in non-target tissues [98,99]. Consequently, achieving controlled release has emerged as a key principle in the design of STING-related NMs. Currently, a variety of intelligent release strategies, including photoresponsive, electrical responsive, and TME-responsive approaches, are being extensively investigated to facilitate the precise release of STING agonists [63,100,101]. Furthermore, it is essential to consider the potential risks associated with individuals who possess highly sensitive immune systems. Autoimmune diseases such as systemic lupus erythematosus and dermatomyositis are frequently characterized by excessive activation of the type I interferon pathway, and since STING functions as an upstream signaling molecule [102,103]. Therefore, therapies involving STING agonists should be approached with caution in such patients, and individualized risk assessments should be incorporated into preclinical evaluations.

Physical Barriers in the TME Limit NMs Delivery and Activation. The high interstitial fluid pressure (IFP) and dense extracellular matrix (ECM) in the TME create a physical barrier that restricts the diffusion and delivery of NMs. To address these delivery challenges, an antiangiogenic agent such as sunitinib can be employed to normalize blood vessels and enhance tissue perfusion [104]. NMs can deliver hyaluronidase in combination to reduce ECM density and IFP, thereby improving tumor penetration of NMs [105]. However, practical applications face several challenges. First, the window for vascular normalization induced by antiangiogenic drugs is typically short, and prolonged use may lead to excessive vascular inhibition, which can adversely affect perfusion. Second, ECM-degrading enzymes are unevenly distributed within the tumor, and excessive degradation may result in structural damage to the tissue and an increased risk of metastasis. Furthermore, whether STING agonists can be efficiently taken up and initiate pathway activation is still limited by the ability of the cells themselves to respond. Therefore, while enhancements to the delivery system can improve drug distribution within tumors, effective strategies to regulate the variability in the activation capabilities of the cGAS-STING pathway across different cell types and microenvironmental conditions are still lacking.

Therefore, beyond physical accessibility, the biological responsiveness of target cells plays a crucial role in determining the overall efficacy of STING-based nanotherapies. Solid tumors frequently contain hypoxic regions, where hypoxia can inhibit STING expression and signal transduction through transcriptional regulation [106]. There was no STING expression in glioma tumor cells, and the expression of STING in gastric cancer and lung cancer was lower than that in normal tissues [[107], [108], [109]]. Similarly, STING expression was low in a variety of tumor cell lines [110,111]. This may be related to epigenetic regulation such as DNA methylation of the STING promoter. Even with the efficient delivery achieved by engineered nanoparticles, tumor cells that lack functional STING pathways are unable to effectively induce immune responses. Consequently, the tumor type and its STING expression profile should be thoroughly considered when designing nanotherapeutic strategies. Indirect activation may be accomplished by targeting APCs rather than tumor cells [112]. Alternatively, a combination of histone deacetylase inhibitors or DNA methyltransferase inhibitors may restore the functional expression of the STING pathway [113,114]. This underscores the importance of engineering approaches to overcome tumor-intrinsic STING deficiencies and optimize therapeutic outcomes. Ionizable lipids within LNPs bind mRNA to form stable complexes and release the payload into the cytoplasm under acidic pH conditions in vivo [115]. This process enables mRNA to activate innate immune responses, exerting antitumor effects. Moreover, independent of mRNA cargo, LNPs possess intrinsic adjuvant properties, promoting CD4^+^ T helper 1 (TH1)-mediated cytokine responses [116]. The TH1 cytokines, such as IFN-γ, facilitate DCs maturation, enhancing their ability to recognize and process PAMPs, thereby amplifying the activity of the cGAS-STING pathway. Efficient targeting is crucial for mRNA delivery [117]. Dietmair et al. [118] employed bispecific antibodies (BsAbs) to improve the targeted delivery of mRNA-LNPs. BsAbs bind to both the PEG on the LNP surface and proteins enriched on target cell surfaces, such as prostate-specific membrane antigen in prostate cancer cells. This strategy achieved efficient mRNA uptake and expression both in vitro and in vivo. Notably, several mRNA-LNP therapeutics optimized with BsAbs for the treatment of solid tumors have progressed to clinical trials in humans [119]. Incorporating surface modifications on LNPs that can specifically recognize and bind to receptors on tumor cell surfaces offers a promising strategy to enhance both the stability and targeted delivery of mRNA [120,121]. Such modifications may include peptides, antibodies, glycans, or ligands, each serving as potential targeting moieties [122,123]. These targeting elements can be combined to further optimize cellular selectivity, uptake efficiency, and tumor penetration capability of LNPs [124]. Additionally, modifications that improve the dispersion and stability of LNPs under physiological conditions may facilitate enhanced tumor cell uptake and promote the activation of the cGAS-STING pathway. To enhance protein expression efficiency, De et al. [125] designed LNPs containing β-amino ester ionizable lipids for the delivery of self-amplifying mRNA. This approach achieved higher and more sustained protein expression at lower doses. Additionally, 1-methylpseudouridine-modified mRNA improved translation efficiency by preventing eIF2α phosphorylation, thereby attenuating immune responses and enhancing protein translation [126]. Ultrasound has also been utilized to assist LNP delivery. Ogawa et al. [127] demonstrated that microbubble-assisted focused ultrasound can transiently open the blood-brain barrier, enabling the delivery of mRNA-LNPs and resulting in rapid expression of exogenous proteins. The integration of materials and drugs must complement existing clinical treatments. For instance, Chio et al. [128] developed a nanomotor containing a STING agonist to activate immune cells in the bladder wall, combining it with bladder infusion therapy to enhance anti-tumor effects.

## 5 Clinical translation challenges

The transition of cGAS-STING pathway-related NMs from laboratory settings to clinical applications continues to encounter several challenges, including scale production and quality control. Most NMs depend on intricate self-organized processes and exhibit high sensitivity to factors such as raw material purity, temperature, and shear stress [129]. To avoid inducing non-specific inflammation when activating the STING pathway, properties such as particle size and loading efficiency must be controlled with precision. The experience gained from mRNA vaccine production, which has established a stable vector production process through standardized microfluidic technology and a high-throughput packaging monitoring system, serves as a valuable reference for the industrialization of STING-related NMs. Furthermore, artificial intelligence (AI) technology can be leveraged to enhance the production of NMs, facilitating material screening, drug screening, and drug release prediction [130]. While AI algorithms offer significant advantages in expediting the design process, experimental validation remains the gold standard for ensuring efficacy and safety.

The second key obstacle is the ambiguity surrounding government regulation. The current drug evaluation system predominantly focuses on drugs or biological products with well-defined chemical structures. In contrast, cGAS-STING NMs typically comprise multiple functional modules, including delivery systems, immune agonists, and surface functionalization modifications, which complicate their integration into the existing single drug evaluation framework. Although the FDA has incorporated RNA-LNP vaccines, such as BNT162b2, into the regulatory system, this pathway has not been systematically extended to STING NMs. Currently, there is no unified standard for preclinical evaluation indicators, indication definitions, toxicity verification criteria, or concomitant diagnostic mechanisms for STING NMs. Particularly given the strong immune activity of these products, accurately defining their therapeutic dose range and safety window is challenging in the absence of an effective toxicity assessment model. Existing clinical studies remain at the stage of local injection of STING agonists to explore their biological activities, and a generalizable administration mode has yet to be established. Therefore, prioritizing the development of regulatory standards for nano-delivery systems is essential to facilitate the clinical application of these products.

The integration of NMs with multiple therapeutic modalities offers opportunities for synergistic effects; however, it is essential to ensure that these modalities complement rather than interfere with each other. For instance, the combination of photothermal effects and immune activation can enhance tumor cell death and immune responses, yet it may also increase the risk of inflammation if not managed appropriately [131]. Effective toxicity management is another critical consideration, as multimodal treatment may exacerbate systemic side effects. Comprehensive preclinical evaluation should investigate the optimal dosage, timing, and sequence of these treatments. Clinical trials should incorporate biomarker-based inclusion criteria and develop diagnostic tools to optimize patient stratification and identify those most likely to benefit from STING-based therapies.

Future research should prioritize the development of more effective nanocarriers, incorporating biomarkers to customize materials and optimize treatment regimens. This effort necessitates interdisciplinary collaboration among materials science, immunology, and oncology. Furthermore, expanding clinical trials with clearly defined endpoints is essential for successfully translating experimental results into clinical outcomes. The transformation of NMs-based cGAS-STING therapies into reliable treatments has the potential to significantly enhance outcomes for cancer patients globally.

## CRediT authorship contribution statement

**Ruicheng Wu:** Writing – original draft, Visualization, Validation, Methodology. **Jie Wang:** Writing – original draft, Visualization. **Dengxiong Li:** Writing – original draft, Visualization. **Ao Li:** Writing – original draft. **Koo Han Yoo:** Project administration. **Zhihong Liu:** Project administration. **Wuran Wei:** Writing – review & editing. **Zhipeng Wang:** Writing – review & editing. **Dechao Feng:** Writing – review & editing, Visualization, Project administration, Methodology, Conceptualization.

## Consent for publication

Not available.

## Ethics approval and consent to participate

Not available.

## Funding

No finding.

## Declaration of competing interest

The authors have no conflicts of interest to declare.

## Data Availability

No data was used for the research described in the article.

## **List of Abbreviations**

DDR =: DNA damage response
cGAMP =: Cyclic GMP–AMP
cGAS =: Cyclic GMP–AMP synthase
dsDNA =: Double-stranded DNA
STING =: Stimulator of interferon genes
TBK1 =: TANK-binding protein kinase 1
IRF3 =: Interferon regulatory factor 3
APCs =: Antigen-presenting cells
NF-κB =: Kappa-light-chain-enhancer of activated B cells
DCs =: Dendritic cells
NMs =: Nanomaterials
PTT =: Photothermal therapy
MNPs =: Metal nanoparticles
CNTs =: Carbon nanotubes
PNPs =: Polymeric nanoparticles
H_2_O_2_ =: Hydrogen peroxide
TME =: Tumor microenvironment
PLGA =: Polylactic-glycolic acid
PEG =: Polyethylene glycol
ICD =: Immunogenic cell death
LNPs =: Lipid nanoparticles
TH1 =: T helper 1
BsAbs =: Bispecific antibodies
